# Susceptibility to SARS-CoV-2 and MERS-CoV in Beagle Dogs

**DOI:** 10.3390/ani13040624

**Published:** 2023-02-10

**Authors:** Kwang-Soo Lyoo, Yoon-Hwan Yeo, Sung-Geun Lee, Minjoo Yeom, Joo-Yeon Lee, Kyung-Chang Kim, Daesub Song

**Affiliations:** 1 Korea Zoonosis Research Institute, Jeonbuk National University, Iksan 54531, Republic of Korea; 2 Department of Veterinary Medicine Virology Laboratory, College of Veterinary Medicine and Research Institute for Veterinary Science, Seoul National University, Seoul 08826, Republic of Korea; 3 Division of Emerging Infectious Disease and Vector Research, Center for Infectious Diseases Research, National Institute of Health, Korea Disease Control and Prevention Agency, Cheongju 28159, Republic of Korea

**Keywords:** SARS-CoV-2, MERS-CoV, dog, susceptibility

## Abstract

**Simple Summary:**

Many studies have evaluated the spread and transmission of SARS-CoV-2 and MERS-CoV in humans; however, the ability of the virus to infect pets, including dogs, has not been fully clarified. Accordingly, in this study, we evaluated the ability of SARS-CoV-2 and MERS-CoV to infect beagle dogs. Our results showed that dogs can be infected by both viruses. Viral shedding into nasal secretions, feces, and urine was observed, and the lung tissues from the dogs inoculated with SARS-CoV-2 or MERS-CoV showed pathological changes, as well as changes in their lactate dehydrogenase levels.

**Abstract:**

The coronavirus disease 19 (COVID-19) pandemic, caused by the severe acute respiratory syndrome, coronavirus 2 (SARS-CoV-2), has resulted in unprecedented challenges to healthcare worldwide. In particular, the anthroponotic transmission of human coronaviruses has become a common concern among pet owners. Here, we experimentally inoculated beagle dogs with SARS-CoV-2 or Middle East respiratory syndrome (MERS-CoV) to compare their susceptibility to and the pathogenicity of these viruses. The dogs in this study exhibited weight loss and increased body temperatures and shed the viruses in their nasal secretions, feces, and urine. Pathologic changes were observed in the lungs of the dogs inoculated with SARS-CoV-2 or MERS-CoV. Additionally, clinical characteristics of SARS-CoV-2, such as increased lactate dehydrogenase levels, were identified in the current study.

## 1. Introduction

Coronavirus disease 2019 (COVID-19) first emerged in China and quickly led to a worldwide pandemic [1]. Although most studies have focused on the pathogenesis of COVID-19 in humans, the zoonotic aspects of the severe acute respiratory syndrome, coronavirus 2 (SARS-CoV-2), have raised public health concerns worldwide [1]. In particular, pet dogs living with individuals affected by COVID-19 have become infected and shown seroconversion in Hong Kong; these initial cases of human-to-animal SARS-CoV-2 transmission have suggested potential anthroponotic issues related to COVID-19 [2]. Accordingly, well-designed studies investigating the susceptibility of dogs to human coronaviruses are urgently needed.

Middle East respiratory syndrome (MERS-CoV), first identified in Saudi Arabia in 2012, belongs to the Betacoronavirus genus, as does SARS-CoV-2, and has a fatality rate of 34.5% owing to severe respiratory illness [3,4]. Although SARS-CoV-2 and MERS-CoV commonly cause lung disease in humans, they have different target receptors, angiotensin-converting enzyme 2 (ACE2) and dipeptidyl peptidase 4 (DPP4), respectively, on the host cell surface [3,4]. DPP4 is less distributed in the upper respiratory tract but is highly expressed in the lower respiratory tract. In contrast, ACE2 is abundant in the upper and lower respiratory tracts. The difference between the two receptors could be expected to have a critical role in the pathogenesis in animal models [5]. Moreover, the transmissibility of MERS-CoV is lower than that of SARS-CoV-2, but the symptoms of MERS-CoV are more severe, leading to higher mortality than SARS-CoV-2 [3,4].

Non-human primates, transgenic mice, and dromedary camels, the natural hosts of the virus, have been shown to be appropriate animal models for MERS-CoV infection through experimental studies using a variety of animal species [6]. Rhesus macaques showed mild to moderate clinical signs and interstitial pneumonia lesions, and the viral antigen was detected in the entire respiratory tract [6,7]. Transgenic mice showed severe weight loss and 100% mortality [6]. Gross and microscopic lesions progressed considerably in the lungs, and the viral antigen was found in various organs. However, no studies have assessed whether MERS-CoV can infect dogs. As MERS-CoV is highly pathogenic among human coronaviruses, this experimental study could demonstrate the potential for reverse zoonosis of the virus.

For SARS-CoV-2, several attempts have been made to select appropriate animal models that can accurately recapitulate the clinical manifestations of COVID-19 [8]. Dogs have also been inoculated with SARS-CoV-2, and limited pathogenesis, such as viral RNA detection in rectal swabs, has been observed [9]. It is considered that further analyses are needed to fully understand the infection mechanism of SARS-CoV-2 because pet dogs share living spaces with humans and are companion animals, and wild-type and variants of SARS-CoV-2 have been detected in pet dogs [2,10,11]. Nevertheless, there are few studies that show that experimentally infected dogs can shed the virus. Accordingly, in this study, we assessed experimental inoculation with the wild-type strain of SARS-CoV-2, which could be a benchmark to compare the susceptibility of other coronaviruses, including MERS-CoV, in dogs.

## 2. Materials and Methods

### 2.1. Viruses

The SARS-CoV-2 (NCCP43326, Wuhan wild-type) and MERS-CoV (National Control Number 1-001-MER-IS-2015001) were provided by the National Culture Collection for Pathogens in Korea and the Korea Disease Control and Prevention Agency, respectively. The viruses were propagated and passaged twice within Vero E6 cells in Dulbecco’s modified Eagle medium (DMEM) with 2% (*v*/*v*) fetal bovine serum (FBS), penicillin (10,000 units/mL), streptomycin (10 mg/mL), and amphotericin B (25 μg/mL) at 37 °C in a humidified CO_2_ incubator. The viral stocks were verified for sterility and gene sequences through whole genome sequencing [12]. The viral procedures were performed in a biosafety level-3 (BL-3) facility at the Korea Zoonosis Research Institute.

### 2.2. Animal Studies

All of the experiments were performed at an animal use biosafety level-3 (ABL-3) facility at the Korea Zoonosis Research Institute, which is certified by the Korea Disease Control and Prevention Agency of the Ministry of Health and Welfare (certification number KCDC-15-3-02). The animal experiments were conducted in accordance with the regulations of the care and use of laboratory animal guidelines of Jeonbuk National University and were approved by the Institutional Animal Care and Use Committee and the experimental protocols requiring biosafety were approved by the Institutional Biosafety Committee of Jeonbuk National University (approval number JBNU 2020–03-002-1). Seven nine month old female beagle dogs were used for this study. Three randomly selected dogs were anesthetized with Zoletil 50 and were inoculated intranasally with SARS-CoV-2 at a dose of 10^5.5^ TCID_50_/mL in 1 mL DMEM. Three other dogs were anesthetized and inoculated intranasally with MERS-CoV at a dose of 10^5.5^ TCID_50_/mL in 1 mL DMEM. One dog was administrated with 1 mL DMEM, intranasally, as a negative control. All of the animals were housed separately in single cages, and their clinical signs and body temperatures were monitored for 7 days post-inoculation. The body weights of all of the dogs were measured for 7 days post-inoculation using an electronic scale. For swab sampling, all of the dogs were anesthetized with Zoletil 50, and nasal, rectal, and urethra swabs were collected from all of the dogs at 0, 3, 5, 6, and 7 days post-inoculation for shedding viral quantification.

### 2.3. Blood Biochemistry and Hematological Examination

For bleeding, the dogs were sedated using an intramuscular injection of medetomidine (0.7 μg/kg, Tomidin^®^, Provet Veterinary Products Ltd., Istanbul, Turkey), and blood samples were collected by jugular vein puncture at 0, 3, 5, 6, and 7 days post-inoculation. The concentrations of alanine aminotransferase (ALT), albumin (ALB), total bilirubin (TBIL), blood urea nitrogen (BUN), alkaline phosphatase (ALP), lactate dehydrogenase (LDH), and creatinine (CREA) were measured in each blood plasma sample using an automated blood chemistry analyzer (VetTest 8008, IDEXX Laboratories Inc., Westbrook, ME, USA).

The complete blood counts were determined using an automated hematological analyzer (Exigo EOS Vet, Boule Medical AB, Spanga, Sweden). The white blood cell (WBC) count, lymphocytes (LYMs), monocytes (MONOs), granulocytes (GRANs), hemoglobin (HGB), hematocrit (HCT), platelet (PLT) count, and red blood cell (RBC) count were measured using whole blood samples. The blood biochemistry and hematological data were compared with each normal range printed out from the blood chemistry analyzer and the hematological analyzer.

### 2.4. Histopathology

All of the dogs were euthanized using an intravenous injection of 0.1 mg/kg pancuronium bromide and 0.1 M KCl at the end of the experiment (7 days post-inoculation). At necropsy, gross lesions were examined in the lungs, pharynx, lymph nodes, spleen, and kidneys, and then tissues were collected and fixed in 4% neutral-buffered formalin for 1 week. The tissues embedded in paraffin blocks were sectioned at a thickness of 4 μm and then mounted onto glass slides. The slides were deparaffinized in xylene and rehydrated through a series of graded 100% ethanol to distilled water and then stained with hematoxylin and eosin. For immunohistochemistry, to detect the SARS-CoV-2 antigen in the lung tissue slides, the deparaffinized and rehydrated slides were blocked for endogenous peroxidase with 3% H_2_O_2_ in phosphate-buffered saline (PBS) for 20 min. The tissue sections were placed in 10 mM citrate buffer (pH 6.0), heated for 1 h, and incubated with SARS Nucleocapsid Protein Antibody (NB100-56576, Novus Biologicals, Centennial, CO, USA) at a 1:200 dilution at 4 °C overnight. For MERS-CoV antigen detection, the tissue sections were digested with proteinase K (P2308, Merck, Darmstadt, Germany) for 30 min at 37 °C and incubated with rabbit polyclonal antiserum against MERS-CoV (Sino Biologicals Inc., Beijing, China) at a 1:1000 dilution at 4 °C overnight. All of the slides were then washed in PBS and incubated with a secondary antibody (RealTM EnvisionTM Detecion system rabbit/mouse, K5007, Dako, Glostrup, Denmark) for 40 min at 37 °C. Color development was performed using 3,3′-diamino-benzidine tetrahydrochloride (DAB; K5007, Dako, Glostrup, Denmark), followed by counterstaining with hematoxylin. Light microscopic examination was performed using a BX53 microscope (Olympus, Tokyo, Japan). In the lung sections, microscopic lesions were evaluated assessing the severity of interstitial, vasculitis and perivasculitis. For the staining of SARS-CoV-2 antigens, the immunohistochemistry assay was performed as described previously [9].

### 2.5. Quantitative Real-Time PCR

To measure the viral loads of SARS-CoV-2 and MERS-CoV in the lung tissues and swab samples, quantitative real-time PCR was performed to detect the N gene of SARS-CoV-2 using a TaqMan Fast Virus 1-Step Master Mix (Thermo Fisher Scientific, Inc., Waltham, MA, USA) and the S2 gene of MERS-CoV using a SensiFAST™ Probe No-ROX One-Step Kit (Bioline, London, UK), as previously described [13,14]. One gram of lung tissue samples from all of the dogs was placed into soft tissue homogenizing CK14 tubes (Precellys, Betin Technologies, Montigny-le-Bretonneux, France) prefilled with ceramic beads and DMEM, and then homogenized using a Bead Blaster 24 (Benchmark Scientific, Sayreville, NJ, USA). The swabs were placed in 1 mL DMEM supplemented with antibiotics and suspended by vortexing. The viral RNA was extracted from the swab samples and the homogenized tissues using a QIAamp viral RNA Mini Kit (Qiagen, Redwood, CA, USA), according to the manufacturer’s protocol. Real-time PCR for each virus was conducted using a CFX96 Touch real-time PCR detection system (Bio-Rad, Hercules, CA, USA).

## 3. Results

### 3.1. Clinical Observations

**Figure 1:**
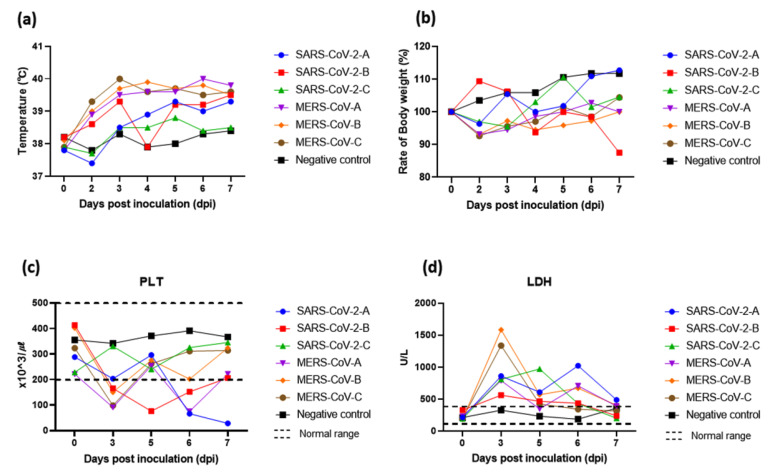
Body temperature changes, weight loss, platelet count (PLT), and lactate dehydrogenase (LDH) levels of dogs inoculated with SARS-CoV-2 or MERS-CoV. Three beagle dogs in each group were inoculated intra-nasally with SARS-CoV-2 (10^5.5^ TCID_50_/mL) or MERS-CoV (10^5.5^ TCID_50_/mL). (**a**) Temperature change; (**b**) weight loss; (**c**) PLT: platelet count; and (**d**) LDH: lactate dehydrogenase.

The Beagle dogs (9 months old) were experimentally inoculated with SARS-CoV-2 or MERS-CoV, and their susceptibility to these human CoVs was assessed. Two out of the three dogs inoculated with SARS-CoV-2 and all three of the dogs inoculated with MERS-CoV showed an elevated body temperature compared with the non-infected dog (Figure 1a; Table S1). Weight loss was also observed in most of the dogs inoculated with SARS-CoV-2 or MERS-CoV until 7 days post-inoculation, whereas the weight of one dog infected with SARS-CoV-2 was recovered 6 days post-inoculation (Figure 1b; Table S1). No other clinical signs were observed in any dogs.

### 3.2. Blood Biochemistry and Hematological Examination

Next, the blood biochemistry and hematological parameters were examined in the dogs inoculated with SARS-CoV-2 or MERS-CoV and compared with those in an uninfected dog. The ALT, ALB, TBIL, BUN, ALP, LDH, CREA, WBC count, LYMs, MONOs, GRANs, PLT count, HGB, HCT, and RBC count were tested using the plasma and whole blood collected at 0, 3, 5, 6, and 7 days post-inoculation (Figures S1 and S2; Tables S2 and S3). The ALT, ALB, BUN, ALP, WBC, LYMs, GRANs, HCT, and RBC were within the normal range for each parameter. In contrast, some of the dogs inoculated with SARS-CoV-2 or MERS-CoV showed PLT levels that had decreased to less than the normal range. In the current study, the PLT counts of the non-infected dogs were between 342 and 391 × 10^3^/µL (normal range: 200–500 × 10^3^/µL), whereas lower counts were observed in the SARS-CoV-2 infected dogs (228, 28, and 76 × 10^3^/µL, respectively) and the MERS-CoV-infected dogs (98, 150, and 75 × 10^3^/µL, respectively; Figure 1c). The MONO counts and HGB levels were somewhat increased in one or two of the infected dogs. The TBIL levels were elevated in some of the infected dogs compared with those in the non-infected dogs.

In the current study of experimental infection, the LDH levels were considerably increased in the dogs infected with SARS-CoV-2 or MERS-CoV; indeed, the LDH levels were increased up to 1.7 fold, 4.2 fold, and 5.5 fold in the three SARS-CoV-2-infected dogs, and they were 2.4, 4.9, and 4.1 fold higher in the three MERS-CoV-infected dogs, whereas the non-infected dog showed LDH levels within the normal range (normal range: 40–400 U/L; Figure 1d).

### 3.3. Viral Loads by Quantitative Real-Time PCR

The Viral RNA loads of SARS-CoV-2 and MERS-CoV in the serum, tonsil, nasal swab, rectal swab, and urethral swab samples of the dogs were determined by real-time PCR (Table 1). In the three dogs infected with SARS-CoV-2, viral RNA was detected in the nasal swabs, rectal swabs, and urethral swabs at 3, 5, 6, and 7 days post-inoculation, whereas MERS-CoV RNA was partially detected in the nasal swabs (three out of three dogs at three days post-inoculation, two out of three dogs at five days post-inoculation, and two out of three dogs at 6 days post-inoculation), rectal swabs (two out of three at three days post-inoculation), and urethral swabs (two out of three dogs at three days post-inoculation). All of the samples were inoculated into Vero E6 cells for the analysis of the viral viability. SARS-CoV-2 was cultivated from some nasal swabs (three out of three dogs at three days post-inoculation, two out of three dogs at five days post-inoculation, and two out of three dogs at six days post-inoculation) and urethral swabs (one out of three dogs at three, five, and six days post-inoculation and two out of three dogs at seven days post-inoculation), but not from the rectal swabs. MERS-CoV was not cultivated from any of the samples.

### 3.4. Lung Pathology

**Figure 2:**
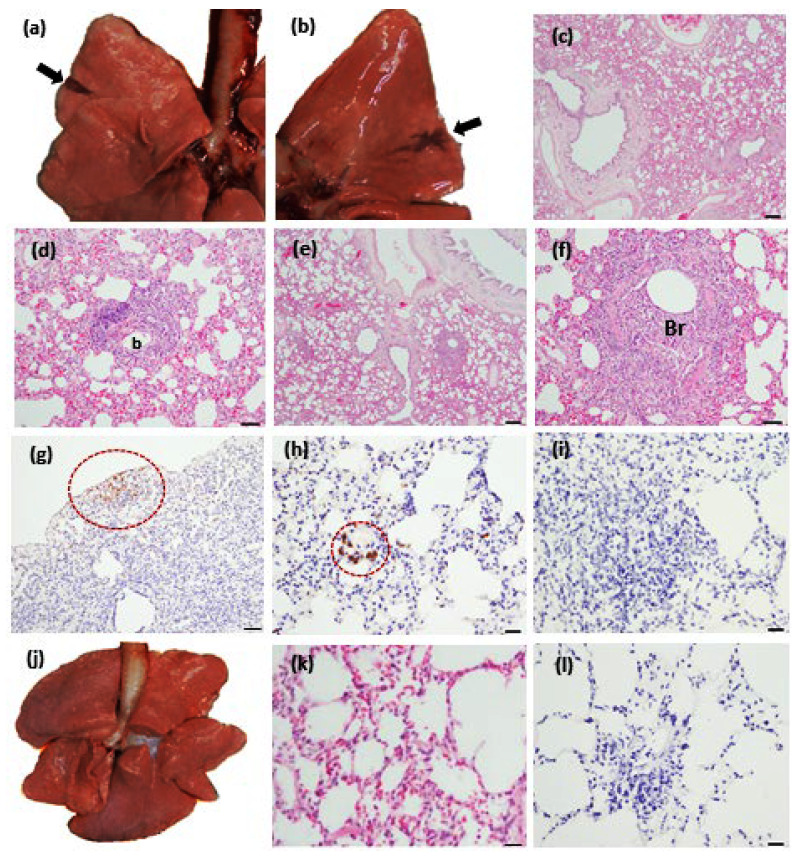
Pathologic changes in the lungs of dogs inoculated with SARS-CoV-2 or MERS-CoV. Pulmonary consolidation (arrowhead) in the right dorsal lobe of a SARS-CoV-2-infected dog (**a**) and in the left dorsal lobe of another SARS-CoV-2-infected dog (**b**). Mild interstitial pneumonia ((**c**); ×40, hematoxylin and eosin stained) and perivascular infiltration of lymphocytes, macrophages, and degenerate neutrophils ((**d**); 273 × 200, (b): blood vessel) in a SARS-CoV-2-infected dog. Mild interstitial pneumonia was observed in a MERS-CoV-infected dog ((**e**); ×40). Focal bronchiolitis (Br) with perivascular infiltration of lymphocytes, macrophages, and degenerate neutrophils was detected ((**f**); ×200). SARS-CoV-2 antigen detection by immunohistochemistry (IHC) in alveolar macrophages and neutrophils in the red circles ((**g**); ×100, bar: 50 µm) ((**h**); ×200; bar: 20 µm) in the lung tissue of a SARS-CoV-2-infected dog. The MERS-CoV antigen was not detected by IHC ((**i**); ×100; bar: 50 µm). Lung (**j**) and histology ((**k**); ×200; hematoxylin-and-eosin stained; bar: 20 µm, (**l**); ×200, IHC; bar: 20 µm) of the negative control dog. S; SARS-CoV-2-infected dogs, M; MERS-CoV-infected dogs, C; negative control dog.

Pathological examinations were performed on the euthanized dogs at the end of the experiment. In the autopsies of the dogs infected with SARS-CoV-2, pulmonary consolidation was observed on each lung surface (Figure 2a,b); however, the other organs were normal. There were no pathologic changes in the organs of the MERS-CoV-infected dogs. The histopathology from the lungs, pharynx, lymph node, spleen, and kidneys from all of the dogs revealed pathologic changes in the lung tissues only. All of the dogs infected with SARS-CoV-2 or MERS-CoV showed similar interstitial pneumonia with mild multifocal peribronchial and perivascular infiltration by lymphocytes, macrophages, and degenerate neutrophils (Figure 2c–f). The immunohistochemistry revealed the presence of the SARS-CoV-2 antigen in the alveolar macrophages and neutrophils in the lung tissue of a SARS-CoV-2 infected dog (SARS-CoV-2-A dog) (Figure 2g,h); however, the MERS-CoV antigen was not detected (Figure 2i).

## 4. Discussion

A number of animal models have been investigated for SARS-CoV-2 infection [8]. Rhesus macaques that mimic the human disease for COVID-19 showed clinical signs and pathogenic lesions in their lungs and have been recognized as a faithful model for studying pathogenesis and vaccine efficacy [8,15]. Golden Syrian hamsters infected with SARS-CoV-2 developed mild clinical signs, but eventually recovered. The animals showed high viral loads in the lungs and pathological changes such as lung consolidation and severe pulmonary hemorrhage [14,15]. Transgenic mice are useful in studying severe SARS-CoV-2 infection. The animals inoculated with SARS-CoV-2 showed weight loss and interstitial pneumonia, and this model can be chosen for testing the vaccine and antiviral drug efficiency [8,15].

Dogs were first used as a model animal for SARS-CoV-2 by a Chinese research group. Their results showed that dogs exhibit low susceptibility to intranasal inoculation with the virus, as demonstrated by partial virus shedding and no viral detection in tissues [9]. Moreover, the overall investigation demonstrated that there is no evidence of SARS-CoV-2 transmission from dogs to dogs or interspecies transmission from dogs to humans [16]. However, we speculated that their data could have been somewhat limited with regard to determining the susceptibility of dogs to SARS-CoV-2. In addition, most pet owners care strongly for their pets and are concerned with the potential of their pets becoming infected [17,18]. Indeed, SARS-CoV-2-specific antibodies were detected, or the clinical signs associated with SARS-CoV-2 infection were observed in pet dogs living in COVID-19-positive households, although there was a lack of reliable data to confirm SARS-CoV-2 transmission from the dogs to people [17,18,19]. Therefore, in this carefully designed study, we aimed to demonstrate the pathogenicity of SARS-CoV-2 in dogs based on the evaluation of multiple parameters, including body weight, temperature, and blood parameters, and to compare the results with those from another human coronavirus, MERS-CoV. Interestingly, our results were not consistent with the previous Chinese study because live SARS-CoV-2 was shed from the nasal discharge and urine of the infected dogs, and some of the dogs exhibited increased body temperature and weight loss.

The necropsy examination showed gross pathology, in the form of focal reddish consolidation, in the lungs. Although the lesions were limited, the pathologic appearance was similar to the gross lung lesion induced by SARS-CoV-2 infection in other species, such as golden Syrian hamsters and the transgenic mice model [14,15]. The lung histopathology showed that viral infection can cause interstitial pneumonia in dogs. The SARS-CoV-2 antigen was detected by IHC in the lung tissue of one dog, but viral RNA was not detected in the lung tissues of all of the infected dogs. It is possible that the real-time PCR assay failed to detect SARS-CoV-2 due to viral RNA degradation before the end of the study, but the viral protein was detectable.

SARS-CoV-2 RNA was detected in all of the nasal and rectal swab samples in this study, and viral viability was even observed in some of the nasal swabs. Therefore, it could be speculated that there is the possibility of SARS-CoV-2 transmission from dogs to humans or other pet animals. In the previous experiment, five beagle dogs (three months old) were intranasally inoculated with SARS-CoV-2, and oropharyngeal and rectal swabs were collected every two days until 14 days post-inoculation [9]. Viral RNA was not detected in any of the oropharyngeal swabs and in only a few rectal swabs (two out of five dogs at two days post-inoculation and one out of four dogs at six days post-inoculation, respectively) [9]. Another study demonstrated that there were no clinical signs, pathologic changes, or SARS-CoV-2 shedding from the experimentally inoculated dogs, but seroconversion was found at 14 days post-inoculation [20]. Furthermore, SARS-CoV-2 RNA was detected in all of the urethral swabs, and some of the collected viruses could be cultivated. SARS-CoV-2 was detected in the urine of a COVID-19 patient, and viral viability also was confirmed in a clinical study [21].

In the MERS-CoV infected dogs, the pathological examinations also showed mild interstitial pneumonia, and MERS-CoV RNA was detected in some of the nasal, rectal, and urethral swab samples, but the viability of MERS-CoV was not determined. Therefore, it is assumed that dogs infected with MERS-CoV may not shed the infectious virus, in contrast to the SARS-CoV-2 infected dogs, and the susceptibility of dogs to MERS-CoV has been demonstrated in the present study.

It is interesting to speculate on the discrepancies between our current results and the results of the previous study, despite the use of the same species and a similar dose of SARS-CoV-2. In veterinary medicine, a dog’s immune system, including the humoral and cellular immune components, is considered mature at six months of age [22]. We used immunologically matured beagle dogs (nine months old) in the current study, whereas immature beagle dogs (three months old) were used in the previous study [9]. It has been generally accepted that COVID-19 symptoms are often weak or absent in pediatric patients compared with those in adult patients [23,24]. Although the reason for this difference is still not clear, it could be speculated to be related to variations in the ACE2 expression levels and in the inflammatory responses associated with immune maturation between adults and children [23,24]. The ACE2 expression levels in the lung epithelial cells are lower in pediatric populations, and adult patients with COVID-19 show more robust pro-inflammatory responses or cytokine storm with worse lung lesions [23,25].

Among the blood parameters, the circulating PLT counts are frequently decreased following virus infections, although the mechanisms of the PLT/virus interaction are multifaceted [26]. LDH is a biomarker that is present in most body tissues and is elevated following tissue damage. Recently, several clinical studies have demonstrated that increased LDH levels are associated with disease severity in patients with COVID-19, suggesting that this parameter may be a useful biomarker for disease progression [27,28]. The LDH levels were markedly altered in the dogs in our study. In a recent report, a predictive model using machine learning algorithms and abundant epidemiological, clinical, and laboratory information was established to identify the prognostic biomarkers for patients with COVID-19 [29]. The model identified three key features, including LDH levels, as important factors for prognostic prediction in patients with COVID-19 [29]. Although the LDH levels were markedly changed in the virus inoculated dogs, some factors, such as the sample collection procedures, may have had an impact on the interpretation of the blood parameter results [30,31]. Therefore, a detailed analysis of blood chemistry and a hematological examination are necessary for animal studies.

## 5. Conclusions

We experimentally inoculated beagle dogs with SARS-CoV-2 or MERS-CoV and evaluated the viral shedding and pathological changes. The viral infection altered the dogs’ body weight, temperature, and pathology, and viral replication was detected with mild lesions in both of the infection groups. Moreover, in the current study, we investigated a canine model of experimental MERS-CoV infection for the first time. The data showed that dogs are susceptible to SARS-CoV-2 and that they may be feasible as another animal model for SARS-CoV-2 research. Hence, further research needs to investigate the pathogenicity of SARS-CoV-2 and MERS-CoV using this dog model.

## Figures and Tables

**Table 1:** Viral RNA detection by real-time PCR and virus cultivation of SARS-CoV-2 or MERS-CoV in tissue samples.

		Lung	Tonsil	Serum					Nasal					Anal					Urethra				
				Days Post-Inoculation					Days Post-Inoculation					Days Post-Inoculation					Days Post-Inoculation				
				0	3	5	6	7	0	3	5	6	7	0	3	5	6	7	0	3	5	6	7
SARS-	A	-	-	-	-	-	-	-	-	2.5 * (+)	3.4 (+)	4.1 (+)	4.5	-	3.7	4.6	4.1	4.9	-	2.7	2.9	3.0	2.4
CoV-2	B	-	-	-	-	-	-	-	-	3.2 (+)	3.6	2.8 (+)	3.4	-	2.2	4.3	4.5	3.7	-	1.4	1.8	2.7	2.3 (+)
	C	-	-	-	-	-	-	-	-	3.4 (+)	3.1 (+)	4.4	4.7	-	3.5	3.9	4.0	3.4	-	2.1 (+)	3.0 (+)	2.4 (+)	1.5 (+)
MERS-	A	-	-	-	-	-	-	-	-	1.6	-	-	-	-	2.3	-	-	-	-	1.5	-	-	-
CoV	B	-	-	-	-	-	-	-	-	2.4	2.1	2.5	-	-	2.1	-	-	-	-	1.7	-	-	-
	C	-	-	-	-	-	-	-	-	1.9	2.2	1.8	-	-	-	-	-	-	-	-	-	-	-
Cont.		-	-	-	-	-	-	-	-	-	-	-	-	-	-	-	-	-	-	-	-	-	-

* Log10 Viral RNA copy numbers/mL, (+): virus cultivation in Vero-E6 cells.

## Data Availability

All the data pertaining to the study are available in the manuscript.

## Supplementary Materials

The following supporting information can be downloaded at: https://www.mdpi.com/article/10.3390/ani13040624/s1, Figure S1: Blood biochemistry levels of dogs infected with SARS-CoV-2 or MERS-CoV; Figure S2: Hematological parameters of dogs infected with SARS-CoV-2 or MERS-CoV; Table S1: Body weight and body temperature of all dogs; Table S2: Blood biochemistry data of all dogs; Table S3: Hematological test data of all dogs.
