# Supplementary material for: Susceptibility to SARS-CoV-2 and MERS-CoV in Beagle Dogs
Source: Animals (Basel). 2023 Feb 10;13(4):624. doi: 10.3390/ani13040624 (PMC9951710; doi:10.3390/ani13040624)
Supplement: Supplementary file 1 [file animals-13-00624-s001.zip › Fig. S2.pdf]

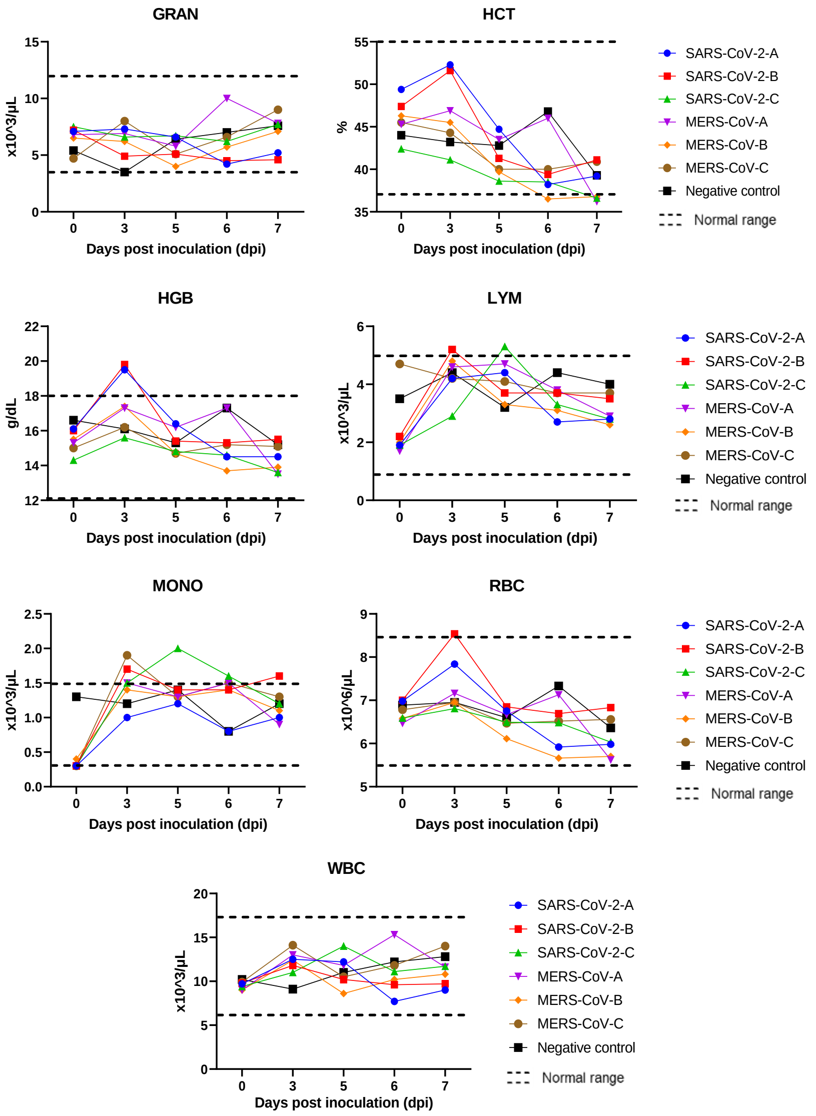
GRAN
x10^3/µL
Days post inoculation (dpi)
HCT
%
Days post inoculation (dpi)
SARS-CoV-2-A
SARS-CoV-2-B
SARS-CoV-2-C
MERS-CoV-A
MERS-CoV-B
MERS-CoV-C
Negative control
Normal range
HGB
g/dL
Days post inoculation (dpi)
LYM
x10^3/µL
Days post inoculation (dpi)
MONO
x10^3/µL
Days post inoculation (dpi)
RBC
x10^6/µL
Days post inoculation (dpi)
WBC
x10^3/µL
Days post inoculation (dpi)
