# Supplementary material for: Susceptibility to SARS-CoV-2 and MERS-CoV in Beagle Dogs
Source: Animals (Basel). 2023 Feb 10;13(4):624. doi: 10.3390/ani13040624 (PMC9951710; doi:10.3390/ani13040624)
Supplement: Supplementary file 1 [file animals-13-00624-s001.zip › Supplementary caption.docx]

Supplementary Materials

**Figure S1. Blood biochemistry levels of dogs infected with SARS-CoV-2 or MERS-CoV.** ALT; Alanine aminotransferase, ALB; albumin, TBIL; total bilirubin, BUN; blood urea nitrogen, ALP; alkaline phosphatase, and CREA; creatinine were measured in blood samples from dogs inoculated with SARS-CoV-2 or MERS-CoV.

**Figure S2. Hematological parameters of dogs infected with SARS-CoV-2 or MERS-CoV.** GRAN**;** granulocyte**,** HCT**;** hematocrit**,** HGB**;** hemoglobin**,** LYM**;** lymphocyte**,** MONO**;** monocyte**,** RBC**;** red blood cell count**,** WBC**;** white blood cell count were measured in blood samples from dogs inoculated with SARS-CoV-2 or MERS-CoV.

**Table S1. Body weight and body temperature of all dogs.**

**Table S2. Blood biochemistry data of all dogs.**

**Table S3. Hematological test data of all dogs.**
