# Supplementary material for: Susceptibility to SARS-CoV-2 and MERS-CoV in Beagle Dogs
Source: Animals (Basel). 2023 Feb 10;13(4):624. doi: 10.3390/ani13040624 (PMC9951710; doi:10.3390/ani13040624)
Supplement: Supplementary file 1 [file animals-13-00624-s001.zip › Table S1.pdf]

Body weight (Kg)								
Group	Dog	days-post-inoculation						
		0	2	3	4	5	6	7
SARS-CoV-2	A	5.50	5.30	5.80	5.50	5.60	6.10	6.20
	B	6.40	7.00	6.80	6.00	6.40	6.30	5.60
	C	6.60	6.40	6.30	6.80	7.30	6.70	6.90
MERS-CoV	A	7.30	6.80	6.90	7.20	7.30	7.50	7.30
	B	7.30	6.80	7.10	6.90	7.00	7.10	7.30
	C	6.80	6.30	6.50	6.60	6.90	6.70	7.10
Negative control		8.50	8.80	9.00	9.00	9.40	9.50	9.50

Body temperatuer (℃)								
Group	Dog	days-post-inoculation						
		0	2	3	4	5	6	7
SARS-CoV-2	A	37.80	37.40	38.50	38.90	39.30	39.00	39.30
	B	38.20	38.60	39.30	37.90	39.20	39.20	39.50
	C	37.90	37.70	38.50	38.50	38.80	38.40	38.50
MERS-CoV	A	37.80	38.90	39.50	39.60	39.60	40.00	39.80
	B	38.10	39.00	39.70	39.90	39.70	39.80	39.50
	C	37.90	39.30	40.00	39.60	39.70	39.50	39.60
Negative control		38.20	37.80	38.30	37.90	38.00	38.30	38.40
