# Supplementary material for: Susceptibility to SARS-CoV-2 and MERS-CoV in Beagle Dogs
Source: Animals (Basel). 2023 Feb 10;13(4):624. doi: 10.3390/ani13040624 (PMC9951710; doi:10.3390/ani13040624)
Supplement: Supplementary file 1 [file animals-13-00624-s001.zip › Table S2.pdf]

Blood biochemistry

Group	Dog	Parameter	days-post-inoculation					Normal range
			0	3	5	6	7	
SARS-CoV-2	A	ALT (U/L)	42	66	50	47	53	10~100
		ALB (g/dL)	3.4	3.3	2.9	3.3	2.7	2.3~4.0
		TBIL (mg/dL)	0.1	1.1	0.2	1.5	0.1	0~0.9
		BUN (mg/dL)	9.82	29	22	25	19	7~27
		ALKP (U/L)	68	101	115	90	96	23~212
		LDH (U/L)	210	859	607	1021	486	40~400
		CREA (mg/dL)	0.6	0.5	0.4	0.4	0.4	0.5~1.8
	B	ALT (U/L)	75	69	65	59	78	10~100
		ALB (g/dL)	2.8	3	2.8	2.9	2.8	2.3~4.0
		TBIL (mg/dL)	0.1	0.1	0.1	0.1	0.1	0~0.9
		BUN (mg/dL)	15	22	19	14	16	7~27
		ALKP (U/L)	95	72	53	59	68	23~212
		LDH (U/L)	325	561	464	434	237	40~400
		CREA (mg/dL)	0.4	0.5	0.4	0.3	0.4	0.5~1.8
	C	ALT (U/L)	53	38	24	65	59	10~100
		ALB (g/dL)	3.3	3.1	2.9	2.8	2.7	2.3~4.0
		TBIL (mg/dL)	0.6	0.6	1.1	0.1	0.1	0~0.9
		BUN (mg/dL)	16.48	28	19	19	22	7~27
		ALKP (U/L)	65	64	50	68	79	23~212
		LDH (U/L)	192	807	971	428	201	40~400
		CREA (mg/dL)	0.6	0.4	0.3	0.3	0.4	0.5~1.8
MERS-CoV	A	ALT (U/L)	48	11	55	49	55	10~100
		ALB (g/dL)	3.3	3.4	2.9	3.3	3	2.3~4.0
		TBIL (mg/dL)	0.06	1.4	0.2	0.6	0.2	0~0.9
		BUN (mg/dL)	13.45	24	17	13	20	7~27
		ALKP (U/L)	73	140	246	133	206	23~212
		LDH (U/L)	231	788	346	705	389	40~400
		CREA (mg/dL)	0.6	0.5	0.5	0.4	0.4	0.5~1.8
	B	ALT (U/L)	42	54	92	64	65	10~100
		ALB (g/dL)	3.4	3.7	3	3	2.9	2.3~4.0
		TBIL (mg/dL)	0.1	2	0.5	0.2	0.1	0~0.9
		BUN (mg/dL)	9.82	22	12	17	13	7~27
		ALKP (U/L)	78	59	63	66	76	23~212
		LDH (U/L)	186	1583	567	669	403	40~400
		CREA (mg/dL)	0.6	0.5	0.6	0.6	0.6	0.5~1.8
	C	ALT (U/L)	29	41	67	59	64	10~100
		ALB (g/dL)	3.2	3.5	2.9	2.9	2.9	2.3~4.0
		TBIL (mg/dL)	0.07	1.5	0.1	0.1	0.1	0~0.9
		BUN (mg/dL)	11.57	22	9	11	19	7~27
		ALKP (U/L)	114	78	117	142	131	23~212
		LDH (U/L)	199	1335	423	340	312	40~400
		CREA (mg/dL)	0.6	0.4	0.3	0.3	0.4	0.5~1.8
Negative control		ALT (U/L)	67	72	87	75	64	10~100
		ALB (g/dL)	2.8	2.9	2.7	2.8	2.8	2.3~4.0
		TBIL (mg/dL)	0.1	0.1	0.1	0.1	0.1	0~0.9
		BUN (mg/dL)	27	16	18	17	16	7~27
		ALKP (U/L)	85	73	78	76	71	23~212
		LDH (U/L)	210	325	231	186	358	40~400
		CREA (mg/dL)	0.4	0.5	0.5	0.6	0.6	0.5~1.8
