# Supplementary material for: Susceptibility to SARS-CoV-2 and MERS-CoV in Beagle Dogs
Source: Animals (Basel). 2023 Feb 10;13(4):624. doi: 10.3390/ani13040624 (PMC9951710; doi:10.3390/ani13040624)
Supplement: Supplementary file 1 [file animals-13-00624-s001.zip › Table S3.pdf]

Hematological test

Group	Dog	Parameter	days-post-inoculation					Normal range
			0	3	5	6	7	
SARS-CoV-2	A	WBC (x10^3/μl)	9.7	12.5	12.2	7.7	9	6.0~17.0
		LYM (x10^3/μl)	1.9	4.2	4.4	2.7	2.8	0.9~5.0
		MONO (x10^3/μl)	0.3	1	1.2	0.8	1	0.3~1.5
		GRAN (x10^3/μl)	7.1	7.3	6.6	4.2	5.2	3.5~12.0
		HGB (g/dL)	16.1	19.5	16.4	14.5	14.5	12.0~18.0
		HCT (%)	49.4	52.3	44.7	38.2	39.2	37.0~55.0
		RBC (x10^6/μl)	6.98	7.84	6.75	5.92	5.98	5.50~8.50
		PLT (x10^3/μl)	288	203	296	66	28	200~500
	B	WBC (x10^3/μl)	9.8	11.8	10.2	9.6	9.7	6.0~17.0
		LYM (x10^3/μl)	2.2	5.2	3.7	3.7	3.5	0.9~5.0
		MONO (x10^3/μl)	0.3	1.7	1.4	1.4	1.6	0.3~1.5
		GRAN (x10^3/μl)	7.2	4.9	5.1	4.5	4.6	3.5~12.0
		HGB (g/dL)	16	19.8	15.4	15.3	15.5	12.0~18.0
		HCT (%)	47.4	51.6	41.3	39.4	41.1	37.0~55.0
		RBC (x10^6/μl)	7	8.54	6.85	6.69	6.83	5.50~8.50
		PLT (x10^3/μl)	413	165	76	152	206	200~500
	C	WBC (x10^3/μl)	9.4	11	14	11.1	11.7	6.0~17.0
		LYM (x10^3/μl)	1.9	2.9	5.3	3.3	2.8	0.9~5.0
		MONO (x10^3/μl)	0.3	1.5	2	1.6	1.2	0.3~1.5
		GRAN (x10^3/μl)	7.5	6.6	6.7	6.2	7.7	3.5~12.0
		HGB (g/dL)	14.3	15.6	14.8	14.6	13.6	12.0~18.0
		HCT (%)	42.4	41.1	38.6	38.5	36.6	37.0~55.0
		RBC (x10^6/μl)	6.59	6.81	6.49	6.48	6.03	5.50~8.50
		PLT (x10^3/μl)	228	330	240	325	345	200~500
MERS	A	WBC (x10^3/μl)	9	13	11.8	15.3	11.6	6.0~17.0
		LYM (x10^3/μl)	1.7	4.6	4.7	3.8	2.9	0.9~5.0
		MONO (x10^3/μl)	0.3	1.5	1.3	1.5	0.9	0.3~1.5
		GRAN (x10^3/μl)	6.8	6.9	5.8	10	7.8	3.5~12.0
		HGB (g/dL)	15.3	17.3	16.2	17.3	13.5	12.0~18.0
		HCT (%)	45.3	46.9	43.5	46	36.2	37.0~55.0
		RBC (x10^6/μl)	6.47	7.16	6.67	7.13	5.62	5.50~8.50
		PLT (x10^3/μl)	221	91	254	75	222	200~500
	B	WBC (x10^3/μl)	9	12.4	8.6	10.2	10.8	6.0~17.0
		LYM (x10^3/μl)	2	4.8	3.3	3.1	2.6	0.9~5.0
		MONO (x10^3/μl)	0.4	1.4	1.3	1.4	1.1	0.3~1.5
		GRAN (x10^3/μl)	6.5	6.2	4	5.7	7.1	3.5~12.0
		HGB (g/dL)	15.5	17.4	14.7	13.7	13.9	12.0~18.0
		HCT (%)	46.3	45.5	39.7	36.5	36.8	37.0~55.0
		RBC (x10^6/μl)	6.58	6.95	6.11	5.66	5.7	5.50~8.50
		PLT (x10^3/μl)	402	150	276	202	324	200~500
	C	WBC (x10^3/μl)	9.9	14.1	10.5	11.8	14	6.0~17.0
		LYM (x10^3/μl)	4.7	4.2	4.1	3.7	3.7	0.9~5.0
		MONO (x10^3/μl)	0.3	1.9	1.3	1.5	1.3	0.3~1.5
		GRAN (x10^3/μl)	4.7	8	5.1	6.6	9	3.5~12.0
		HGB (g/dL)	15	16.2	14.7	15.2	15.1	12.0~18.0
		HCT (%)	45.5	44.3	40	40	40.9	37.0~55.0
		RBC (x10^6/μl)	6.78	6.95	6.47	6.52	6.56	5.50~8.50
		PLT (x10^3/μl)	323	98	262	311	314	200~500
Negative control		WBC (x10^3/μl)	10.2	9.1	11	12.2	12.8	6.0~17.0
		LYM (x10^3/μl)	3.5	4.4	3.2	4.4	4	0.9~5.0
		MONO (x10^3/μl)	1.3	1.2	1.4	0.8	1.2	0.3~1.5
		GRAN (x10^3/μl)	5.4	3.5	6.4	7	7.6	3.5~12.0
		HGB (g/dL)	16.6	16.1	15.3	17.3	15.2	12.0~18.0
		HCT (%)	44	43.2	42.8	46.8	39.3	37.0~55.0
		RBC (x10^6/μl)	6.89	6.95	6.6	7.33	6.36	5.50~8.50
		PLT (x10^3/μl)	355	342	371	391	367	200~500
