# Supplementary figures and images for: Susceptibility to SARS-CoV-2 and MERS-CoV in Beagle Dogs
Source: Animals (Basel). 2023 Feb 10;13(4):624. doi: 10.3390/ani13040624 (PMC9951710; doi:10.3390/ani13040624)

ALT

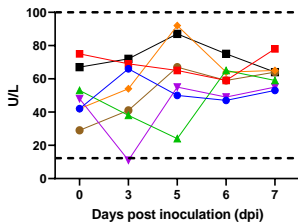

ALB

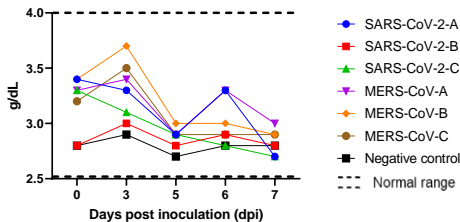

TBIL

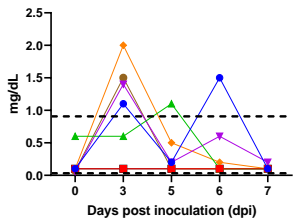

BUN

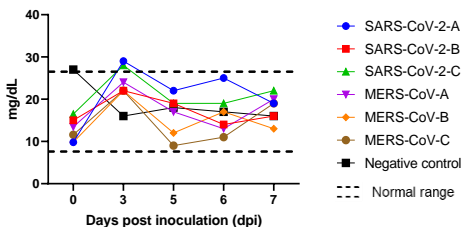

ALP

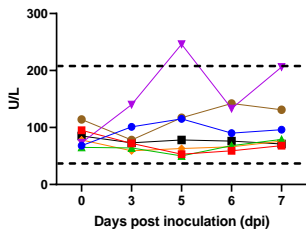

CREA

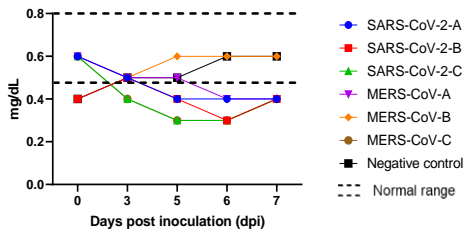

Supplement: Supplementary file 1 [file animals-13-00624-s001.zip › Fig. S1.pdf]
